# Effect of bisphosphonate initiation at week 2 versus week 12 on short-term functional recovery after femoral neck fracture: a randomized controlled trial

**DOI:** 10.1007/s11657-017-0321-8

**Published:** 2017-03-10

**Authors:** Aasis Unnanuntana, Panai Laohaprasitiporn, Atthakorn Jarusriwanna

**Affiliations:** Department of Orthopaedic Surgery, Faculty of Medicine Siriraj Hospital, Mahidol University, 2 Prannok Road, Bangkoknoi, Bangkok, 10700 Thailand

**Keywords:** Randomized controlled trial, Femoral neck fracture, Bisphosphonate, Risedronate, Hemiarthroplasty

## Abstract

**Summary:**

The appropriate time to initiate bisphosphonate treatment after a fragility fracture has not yet been established. In this study, we found no significant differences in short-term functional recovery between femoral neck fracture patients who received bisphosphonate treatment at 2 versus 12 weeks after hemiarthroplasty.

**Introduction:**

Bisphosphonate is the mainstay therapy for prevention and treatment of osteoporosis. The aim of this study was to investigate the effect of bisphosphonate initiation on short-term functional recovery in femoral neck fracture patients at 2 versus 12 weeks after hemiarthroplasty.

**Methods:**

One hundred patients were randomly allocated into two groups in a parallel group designed, randomized, controlled trial. Both groups received risedronate 35 mg/week at either 2 or 12 weeks after hemiarthroplasty. All patients received calcium and vitamin D supplementation. Functional recovery was assessed by de Morton Mobility Index, Barthel Index, EuroQol 5D, visual analog scale, 2-min walk test, and timed get-up-and-go test at 2 weeks, 3 months, and 1 year after surgery.

**Results:**

At the 3-month follow-up, all functional outcome measures showed significant improvement in both groups. There were no statistically significant differences in any of the functional outcomes between groups at both the 3-month and 1-year follow-ups. Although patients who received bisphosphonate initiation at week 2 had lower serum calcium level at 3 months and more overall adverse events than patients in the week 12 group, no patients in either group discontinued their prescribed medications.

**Conclusions:**

While underpowered, the findings of this study suggest that there were no significant differences in short-term functional recovery or significant adverse events between the two bisphosphonate groups. Thus, the initiation of bisphosphonate therapy may be considered as early as 2 weeks after femoral neck fracture. It is important that low serum calcium and vitamin D status must be corrected with calcium and vitamin D supplementation prior to or at the time of bisphosphonate initiation.

**Clinical trial registration number:**

This study was registered in the database via the Protocol Registration and Results System (PRS) (NCT02148848).

**Electronic supplementary material:**

The online version of this article (doi:10.1007/s11657-017-0321-8) contains supplementary material, which is available to authorized users.

## Introduction

Hip fracture is a common osteoporotic fracture among the elderly and is a major public health concern worldwide [1]. It was estimated that the total number of hip fractures in persons 50 years of age or older will increase worldwide from 1.7 million in 1990 to 6.3 million in 2050 [2]. With changes in the demographics of populations around the world and more elderly living in developing countries, it is predicted that half of all hip fractures will occur in Asia [3]. Hip fracture among the elderly results in serious health consequences with significant mortality and significant loss of mobility and independence among those who survive [4, 5]. It was reported that only about 40% of hip fracture patients could return to their preinjury level of walking [6]. In addition, the risk of subsequent hip fracture increases dramatically in this population. Patients with history of fracture have an 86% increase in the risk of sustaining another fracture [7], and half of hip fracture patients have suffered prior fragility fractures [8]. Furthermore, the cost of treating a hip fracture patient is high, with costs being approximately three times higher than those incurred when caring for a patient without a fracture [9, 10]. It is, therefore, essential to develop an effective prevention program and to initiate osteoporosis treatment in these patients to prevent or minimize the chance of future fracture. Although many strategies have been implemented to manage osteoporosis patients, the rate of osteoporosis evaluation and treatment remains low [11]. This may suggest that some physicians had insufficient participation in the prevention of their patients’ secondary fracture. If an intervention with good efficacy can be implemented soon after a fragility fracture has been diagnosed, it is plausible to assume that the rate of osteoporosis treatment will increase.

Among all osteoporosis drugs, bisphosphonates are the most commonly prescribed medication [12]. Bisphosphonates are structurally related to pyrophosphates, which are incorporated into the bone matrix by binding to calcium and hydroxyapatite [13]. Once administered, bisphosphonates inhibit bone resorption by blocking farnesyl diphosphate synthase in the mevalonate pathway, which, in turn, leads to osteoclast apoptosis [14]. Studies have shown that bisphosphonates reduce the incidence of vertebral and nonvertebral fractures (including hip fractures) when used to treat postmenopausal osteoporosis [15].

Hemiarthroplasty is a common surgical procedure for treatment of displaced femoral neck fracture in the elderly [16], because it provides stable fixation and allows for early mobilization of the patient. Femoral neck fracture patients that are treated with arthroplasty provide a good opportunity for orthopedic surgeons to initiate osteoporosis treatment, because there is no concern regarding fracture healing in these patients. However, some physicians have expressed concern regarding the initiation of bisphosphonate prior to correcting low calcium and vitamin D status—a condition that is very common in hip fracture patients [17]. Maalouf et al. [18] reported three cases of bisphosphonate-induced hypocalcemia and concluded that this type of hypocalcemia could develop in patients with unrecognized hypoparathyroidism, impaired renal function, and vitamin D deficiency. We hypothesized that early initiation of bisphosphonate treatment would not affect postoperative functional outcomes and would not increase the rate of adverse effect of bisphosphonate in this group of patients. Accordingly, the objective of this randomized controlled study was to investigate the effect of bisphosphonate initiation on short-term functional recovery in femoral neck fracture patients at 2 versus 12 weeks after hemiarthroplasty.

## Methods

The protocol and consent forms used in this study were approved by the Siriraj Institutional Review Board (SIRB), Faculty of Medicine Siriraj Hospital, Mahidol University. This study was registered in the ClinicalTrials.gov database via the Protocol Registration and Results System (PRS) (NCT02148848). A detailed informed consent form was signed by each participating patient, and all patient information was kept confidential. The study design and reporting format were based on Consolidated Standards of Reporting Trials (CONSORT) principles.

We prospectively screened patients within 2 weeks after surgery who underwent hemiarthroplasty for femoral neck fracture at Siriraj Hospital during the June 2013 to June 2015 study period. Siriraj Hospital is Thailand’s largest university-based national tertiary referral center. Exclusion criteria were as follows: pathologic fracture; multiple fractures; patients with history of abnormalities of the esophagus that delay esophageal emptying, such as stricture or achalasia; patients who were unable to remain upright for 30 min after dosing; patients with hypocalcemia (serum calcium <8.5 mg/dL), severe vitamin D deficiency (serum 25-hydroxyvitamin D (25(OH)D) <10 ng/mL), or metabolic bone diseases other than postmenopausal osteoporosis; patients with estimated glomerular filtration rate (eGFR) less than 35 mL/min/1.73 m^2^ (eGFR was calculated by using the Chronic Kidney Disease Epidemiology Collaboration (CKD-EPI) equation); patients with postoperative complications that required modification of the postoperative rehabilitation protocol; and patients with severe cognitive impairment. Patients were also excluded if they had taken estrogen (or its analogs) or anabolic steroids within the preceding 6 months. Prior use of any bisphosphonate or anabolic agent within 1 year of the study or for ≥2 years within any 5-year period was also a reason for exclusion. Lastly, any patient who was treated with glucocorticoids (≥5 mg/day of prednisolone or its equivalent) within the past 6 months was also not approved for participation in this study.

Patients who met all eligibility criteria were enrolled and sequentially assigned an allocation number into one of the two groups. Randomization was stratified by mode of fixation (cemented or cementless) to balance potential prognostic characteristics between groups. The randomization sequence was concealed prior to enrollment. Although patients and physicians were not blinded to the given intervention, the research assistant who collected the study data was blinded to which treatment arm the patient had been assigned. Patients were allocated to starting bisphosphonate at either week 2 or week 12 postoperatively by a computer-generated blocked randomization scheme using block sizes of 2 and 4. Block randomization was performed according to the type of treatment. Participants were randomized according to the study protocol, with 49 patients allocated to the bisphosphonate initiation at week 2 group and 51 patients allocated to the bisphosphonate initiation at week 12 group. Patients in the bisphosphonate initiation at week 2 group received risedronate (Actonel; Procter & Gamble Pharmaceuticals, Cincinnati, OH, USA) 35 mg/week at 2 weeks after surgery. Patients in the bisphosphonate initiation at week 12 group received risedronate 35 mg/week at 12 weeks postoperatively. For ethical reasons, bisphosphonates were deferred for no longer than 3 months in the bisphosphonate initiation at week 12 group. Risedronate was taken with a full glass of water (6–8 oz) after fasting and upon arising in the morning. The patient was asked to remain in an upright position for approximately 30 min before taking first food or beverage of the day. In addition to the study medication, all patients were instructed to take calcium (1000 mg/day) and vitamin D supplementation. Vitamin D supplementation was given according to the patient’s baseline serum 25(OH)D level. Patients received vitamin D2 dosages, as follows: Those with a 25(OH)D level of 30–40 ng/mL received 20,000 IU of vitamin D2 per week; those with a 25(OH)D level of 20–30 ng/mL received 40,000 IU per week; and those with a 25(OH)D level of <20 ng/mL received 60,000 IU per week. Patients who had a 25(OH)D level of >40 ng/mL were not given additional vitamin D2 supplementation. At the conclusion of the study, participants were advised to continue calcium and vitamin D supplementation indefinitely, and that anti-osteoporosis therapies should be considered or continued at their physician’s discretion.

## Surgery and postoperative rehabilitation protocol

All operations were performed via a posterior approach. The prostheses used in this study were either cementless, metaphyseal fit stem-type, or cemented, polished-type femoral component. Decision regarding type of fixation was based on intraoperative stability of the implant. Adequacy of stability for cementless implant was evaluated and determined from stability status in three dimensions: axial, rotation, and flexion extension. If adequate stability of a cementless implant could not be achieved during surgery, a cemented stem would then be implanted. All patients in both groups were treated using the same postoperative protocol. Physical therapy was started 1 day after the procedure. The physical therapy protocol consisted of early mobilization, including bed exercises and training for ambulation. Bed exercises focused on breathing and the postural lower limbs and were performed using both legs. Exercises included ankle pumps and buttock contractions. Ambulation training was divided into the following rehabilitation milestones: (1) transferring unassisted in and out of bed and (2) being able to stand and walk with an assisting device on a level surface. Patients were allowed to bear full weight, regardless of whether they underwent cemented or cementless fixation. Once they had achieved their physical therapy goals, patients were discharged from the hospital to their home or a rehabilitation facility. It should be noted that the physical therapy goals were adjusted to the needs of each patient based on his/her preoperative ambulatory status and household environment. Physical therapy was given during the hospital admission. Each patient participated in a 1-h physical therapy session each weekday on the patient’s hospital unit that consisted of exercise training and practice. After discharge from the hospital, patients were instructed to continue exercising by walking on a level surface. Pain medications included acetaminophen, short-acting opioids, and intravenous opioids (only during hospital admission). The goal was to keep pain at no higher than 2 to 3 of 10, as measured by visual analog scale.

## Assessment of outcomes

Functional recovery was assessed by de Morton Mobility Index (DEMMI), Barthel Index, EuroQol 5D (EQ-5D), visual analog scale, 2-min walk test, and timed get-up-and-go test. All of these functional outcomes were tested at three intervals for purposes of comparison at the following time points: 2 weeks (as baseline data), 3 months, and 1 year after surgery. We used the change in the DEMMI score between baseline and the 3-month follow-up as the primary outcome, with changes in other scales regarded as being secondary outcomes.

### de Morton Mobility Index

DEMMI is a method to evaluate level of patient mobility [19]. DEMMI was validated among patients in rehabilitation following hip fracture and is freely available for download from www.demmi.org.au [20]. DEMMI is administered to observe physical performance and consists of 15 hierarchical mobility items, including three beds, three chairs, four static balance, two walking, and three dynamic balance items. Each mobility item is measured on a two (able/unable) or three (able/partial/unable) point scale [19]. The sum score, ranging from 0 to 58, is then converted to an interval score that ranges from 0 to 100, where 0 represents no or very poor mobility and 100 indicates high levels of independent mobility [20].

### Barthel Index

The Barthel Index is an ordinal scale for the functional assessment of disability that has been widely used in individuals receiving in-patient rehabilitation, mainly in stroke outcome research [21–23]. It has also been used in and validated for hip fractures in the elderly population [24]. Barthel Index comprises ten variables that describe activities of daily living (ADLs) and mobility, including the presence or absence of fecal and/or urinary incontinence, help needed with grooming, toilet use, feeding, transfers, walking, dressing, climbing stairs, and bathing. Each performance item is rated on a scale with a given number of points assigned to each level or ranking. A higher score is associated with a greater likelihood of being able to live at home with a degree of independence following discharge from the hospital [21]. Barthel Index has high inter-rater reliability (0.95) and test-retest reliability (0.89), as well as high correlations (0.74–0.8) with other measures of physical disability [25].

### EQ-5D

The EQ-5D was designed for self-completion by the participant. This clinical tool is composed of two parts: the EQ-5D-5L utility score (EQ-US) and the EQ visual analog scale (EQ-VAS). In this study, we used only the EQ-VAS, which is a self-evaluated scale. Patients were asked to score their health status on a visual analog scale that ranged from 0 to 100. The top score of the scale (100) represents the best imaginable health state, while the bottom (0) represents the worst imaginable state. EuroQol is a reliable and valid instrument for assessing health-related quality of life in elderly patients with femoral neck fracture [26].

### Visual analog scale

Overall pain was evaluated using visual analog scale. Patients were asked to rate their pain during the past 24 h on a visual analog scale that ranged from 0 to 10, with a higher score reflecting a higher level of pain.

### Two-minute walk test and timed get-up-and-go test

Functional evaluation was conducted using two performance-based tests: 2-min walk test and timed get-up-and-go test. The results of these two tests were recorded at baseline and at two postoperative follow-ups by one of our research assistants who was blinded to the treatment group. For the 2-min walk test, patients were asked to walk up and down a designated corridor for 2 min. Patients were instructed to walk at their normal pace and to turn around at the ends of the corridor without stopping [27]. Results were recorded as total distance walked in meters.

For the timed get-up-and-go test, patients were instructed to rise from a high-seated chair, walk at a safe and comfortable pace to a mark 3 m away, and return to a sitting position with their backs against the chair [28]. Patients were permitted to use their arms when rising from and returning to a seated position. A stopwatch was used to measure the time to complete this activity to the nearest one tenth of a second. Patients were asked to perform this task three times, and the average time was used for analysis.

## Statistical analysis

Statistical power was determined from our primary outcome, which was change in DEMMI score between baseline and the 3-month follow-up. A previous investigation by de Morton et al. [20] found that the standard deviation of the DEMMI score in hip fracture patients was 8.9. Based on the results of that study, we designed the current study to determine a six-point difference, which is the minimally clinically important difference in DEMMI score [20] between the two groups, with a standard deviation of 8.9. Power analysis and sample size calculations indicated that a sample size of 39 patients per group would provide 80% statistical power to detect this effect size between the two groups (alpha = 0.05, beta = 0.20) using Student’s *t* test. Since recruitment was increased by 10% to allow for loss to follow-up, a total of 43 patients per group were required for this study.

Baseline characteristics and the results of both groups were compared using Pearson’s chi-squared or Fisher’s exact test for categorical variables and Student’s *t* test for continuous variables. Kolmogorov-Smirnov test was used to test normality of distribution. One-way repeated measures ANOVA was used to assess the effect of time on the change in each outcome measure for each patient group. Data are presented as number, number (%), or mean ± standard deviation. An intention-to-treat analysis was conducted based on the two initial randomization groups. All analyses were performed using PASW Statistics for Windows, version 18.0 (SPSS, Inc., Chicago, IL, USA). A *p* value <0.05 was regarded as being statistically significant.

## Results

**Fig. 1:**
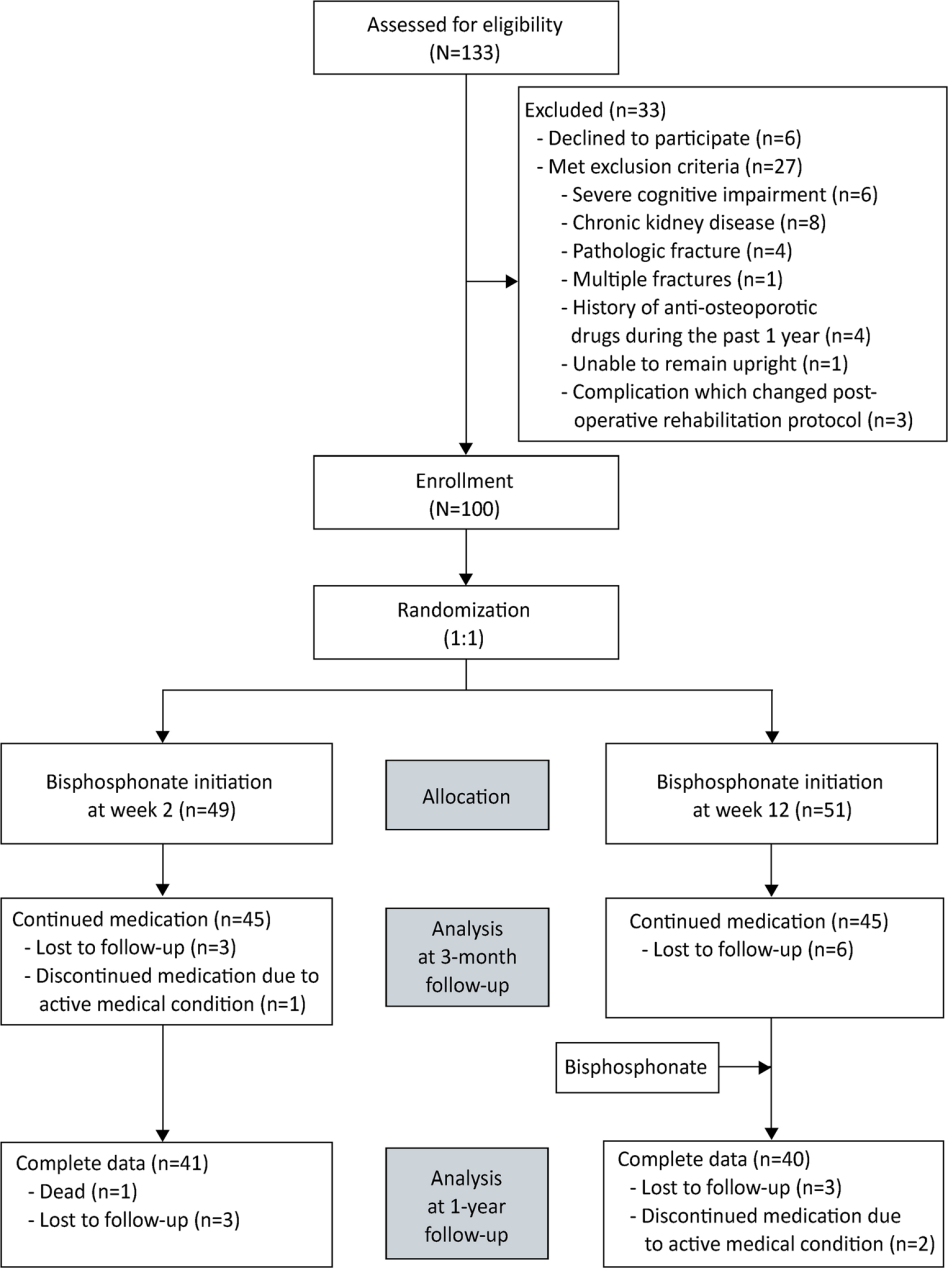
Consolidated Standards of Reporting Trials (CONSORT) diagram showing the flow of patients in the study. The group with bisphosphonate initiation at week 2 received bisphosphonate at 2 weeks after surgery. The group with bisphosphonate initiation at 12 weeks received bisphosphonate at 12 weeks after surgery

A total of 133 patients were screened for eligibility during the study period. Thirty three of those patients were excluded, as follows: six patients for declining to participate in the study, six patients who had severe cognitive impairment, eight patients with eGFR less than 35 mL/min/1.73 m^2^, four patients with pathologic fracture, one patient with multiple fractures, four patients who had history of taking anti-osteoporotic drugs during the past 1 year, one patient who was unable to remain upright for 30 min, and three patients with postoperative complications that required modification of the postoperative rehabilitation protocol (one with a postoperative periprosthetic femoral fracture and two with active medical conditions). The remaining 100 patients were enrolled in the study. Participants were randomized according to the study protocol, with 49 patients being allocated to the bisphosphonate initiation at week 2 group and 51 patients allocated to the bisphosphonate initiation at week 12 group. One patient in the week 2 group died at 9 months after surgery due to an active lung infection. A total of 15 patients were lost during the follow-up period, with 6 and 9 of those patients belonging to the week 2 and week 12 groups, respectively. Three patients discontinued medication after the randomization process due to their active medical condition (one and two patients in the week 2 and week 12 groups, respectively). Ninety patients (90%) and 81 patients (81%) completed the study with data available for analysis at 3 months and 1 year, respectively (Fig. 1).

Demographic and baseline characteristics of each treatment group are provided in Table 1. Mean age in our study was 76.6 years, and most (80%) subjects were women. A majority of study participants received bipolar hemiarthroplasty using cementless femoral component. Approximately 73% of patients could walk without the use of any assisting device before fracture. The average length of stay was similar between the week 2 and week 12 groups (*p* = 0.534). When comparing data between the two groups, there were no differences in any demographic or clinical characteristics, including baseline serum calcium, serum 25(OH)D, and bone mineral density (*p* > 0.05 for all comparisons) (Table 1). When comparing data between patients who completed and did not complete the study at 1 year, there were no significant differences in demographic and clinical characteristics between patients available for analysis and those who did not complete the study at 1 year, except that there were less female patients in the group that did not complete the study at 1 year (*p* = 0.021, electronic supplementary file table).

**Table 1:** Patient demographic and clinical characteristics

Clinical variables	BIS initiation at week 2 (*n* = 49)	BIS initiation at week 12 (*n* = 51)	*p* value
Age (years)	77.3 ± 8.1	75.8 ± 8.3	0.381
Female gender	39 (79.6%)	41 (80.4%)	0.920
Right side	19 (38.8%)	27 (52.9%)	0.155
Body mass index (kg/m^2^)	22.4 ± 3.5	24.0 ± 5.2	0.061
Charlson comorbidity index 0–1 2–3 >3	43 (87.8%) 6 (12.2%) 0 (0.0%)	43 (84.3%) 7 (13.7%) 1 (2.0%)	0.595
Cementless femoral component	43 (87.8%)	43 (84.3%)	0.620
Preoperative ambulatory status With assisting device Without assisting device	9 (18.4%) 40 (81.6%)	18 (35.3%) 33 (64.7%)	0.057
Length of hospital stay (days)	12.7 ± 7.7	10.9 ± 4.8	0.534
Estimated glomerular filtration rate (mL/min/1.73 m^2^)	72.7 ± 20.3	70.2 ± 20.8	0.461
Serum calcium level (mg/dL)	9.0 ± 0.5	9.0 ± 0.6	0.925
Serum 25(OH)D level (ng/mL)	22.8 ± 11.4	21.7 ± 10.3	0.699
Baseline bone mineral density (g/cm^2^) Lumbar spine Femoral neck Total hip	0.958 ± 0.216 0.635 ± 0.112 0.674 ± 0.127	0.936 ± 0.193 0.640 ± 0.139 0.678 ± 0.120	0.596 0.507 0.865

Data presented as number (%) or mean ± standard deviation. *p* value <0.05 indicates statistical significance. Estimated glomerular filtration rate was calculated using the Chronic Kidney Disease Epidemiology Collaboration (CKD-EPI) equation

*BIS* bisphosphonate, *25(OH)D* 25-hydroxyvitamin D

At the 3-month postoperative follow-up, functional outcome in both groups improved significantly (Table 2). The change in scores for DEMMI, Barthel Index, EQ-VAS, and visual analog scale from baseline to 3 months after surgery was similar between the two treatment groups (*p* > 0.05). For the 2-min walk test at the 3-month postoperative follow-up, patients in both groups could walk approximately 2.3 times longer than the distance they could walk at 2 weeks after surgery. For the timed get-up-and-go test at the 3-month postoperative follow-up, patients in both groups could perform this test approximately two times faster than the average recorded time at 2 weeks after hemiarthroplasty. There were no significant differences between the week 2 and week 12 groups for either the 2-min walk test or the timed get-up-and-go test (Table 2).

**Table 2:** Changes in outcome measures between baseline and the 3-month follow-up

Outcome measures	BIS initiation at week 2 (*n* = 45)	BIS initiation at week 12 (*n* = 45)	*p* value
de Morton Mobility Index Baseline 3-month follow-up Change in scores	39.0 ± 8.5 61.0 ± 17.9 22.0 ± 14.1	37.8 ± 9.8 62.7 ± 19.0 25.0 ± 15.7	0.522 0.327 0.345
Barthel Index Baseline 3-month follow-up Change in scores	57.6 ± 15.1 86.4 ± 13.9 28.8 ± 15.1	59.9 ± 16.8 86.6 ± 14.9 26.7 ± 13.1	0.491 0.668 0.459
EuroQol visual analog scale Baseline 3-month follow-up Change in scores	61.0 ± 15.3 75.5 ± 15.9 14.5 ± 16.0	63.1 ± 15.9 78.8 ± 13.9 15.7 ± 16.5	0.527 0.406 0.732
Visual analog scale Baseline 3-month follow-up Change in scores	2.7 ± 2.3 1.1 ± 1.9 −1.8 ± 2.6	2.7 ± 2.5 1.1 ± 1.3 −1.6 ± 2.3	0.930 0.977 0.931
Outcome measures	BIS initiation at week 2 (*n* = 44)	BIS initiation at week 12 (*n* = 42)	*p* value
Two-minute walking test (m) Baseline 3-month follow-up Change in scores	17.4 ± 18.2 40.5 ± 20.9 23.1 ± 23.0	15.9 ± 8.7 38.8 ± 25.3 22.9 ± 19.8	0.621 0.885 0.971
Timed get-up-and-go test (s) Baseline 3-month follow-up Change in scores	71.0 ± 40.3 28.3 ± 16.8 −42.7 ± 33.4	66.1 ± 46.1 32.5 ± 22.1 −33.5 ± 42.9	0.600 0.166 0.272

Data presented as mean ± standard deviation; *p* value <0.05 indicates statistical significance

*BIS* bisphosphonate

Serum calcium and vitamin D levels at baseline and at 3, 6, and 12 months after calcium and vitamin D supplementation for both groups are shown in Table 3. Serum 25(OH)D increased significantly in both groups after 3 months of vitamin D supplementation (mean serum 25(OH)D at 3 months postoperatively = 33.1 and 33.1 ng/mL for the week 2 and week 12 groups, respectively). There was no statistically significant difference in postoperative 25(OH)D level between groups at all time points (*p* = 0.730–0.966). Serum calcium at 3 months after surgery was higher in the week 12 group (mean serum calcium at 3 months postoperatively = 9.1 and 9.4 mg/dL for the week 2 and week 12 groups, respectively; *p* = 0.008). However, there were no differences in serum calcium at 6 and 12 months after surgery between the two groups. Two patients in the week 2 group complained of myalgia, and one had flu-like symptoms, while no patients in the week 12 group reported these symptoms (Table 4). The overall rate of adverse events was higher in the week 2 group than in the week 12 group, but the difference was not statistically significant (16.3 vs. 5.9%, respectively; *p* = 0.095) (Table 4). No patients in either group discontinued any study medications or supplements due to these adverse reactions.

**Table 3:** Changes in serum calcium and serum vitamin D levels after calcium and vitamin D supplementation

Laboratory test	BIS initiation at week 2 (*n* = 41)	BIS initiation at week 12 (*n* = 40)	*p* value
Serum calcium (mg/dL) Baseline 3-month follow-up 6-month follow-up 12-month follow-up	8.9 ± 0.5 9.1 ± 0.4 9.2 ± 0.4 9.1 ± 0.4	9.0 ± 0.6 9.4 ± 0.4 9.2 ± 0.3 9.2 ± 0.4	0.895 0.008 0.757 0.441
Serum 25-hydroxyvitamin D (ng/mL) Baseline 3-month follow-up 6-month follow-up 12-month follow-up	21.9 ± 9.7 33.1 ± 9.1 35.1 ± 8.2 34.8 ± 6.2	22.9 ± 9.4 33.1 ± 9.0 34.5 ± 6.9 34.5 ± 7.5	0.672 0.966 0.730 0.866

Data presented as mean ± standard deviation. *p* value <0.05 indicates statistical significance

*BIS* bisphosphonate

**Table 4:** Type and frequency of adverse events among the study population

Adverse events	BIS initiation at week 2 (*n* = 49)	BIS initiation at week 12 (*n* = 51)	*p* value
Nausea/vomiting	1	1	0.095
Constipation	–	1	
Diarrhea	1	–	
Myalgia	2	–	
Flu-like symptoms	1	–	
Muscle cramp	–	1	
Hearing impairment	1	–	
Deep vein thrombosis	1	–	
Ischemic stroke	1	–	
Total number of adverse events	8 (16.3%)	3 (5.9%)	

Data presented as number or number (%); *p* value <0.05 indicates statistical significance

*BIS* bisphosphonate

**Fig. 2:**
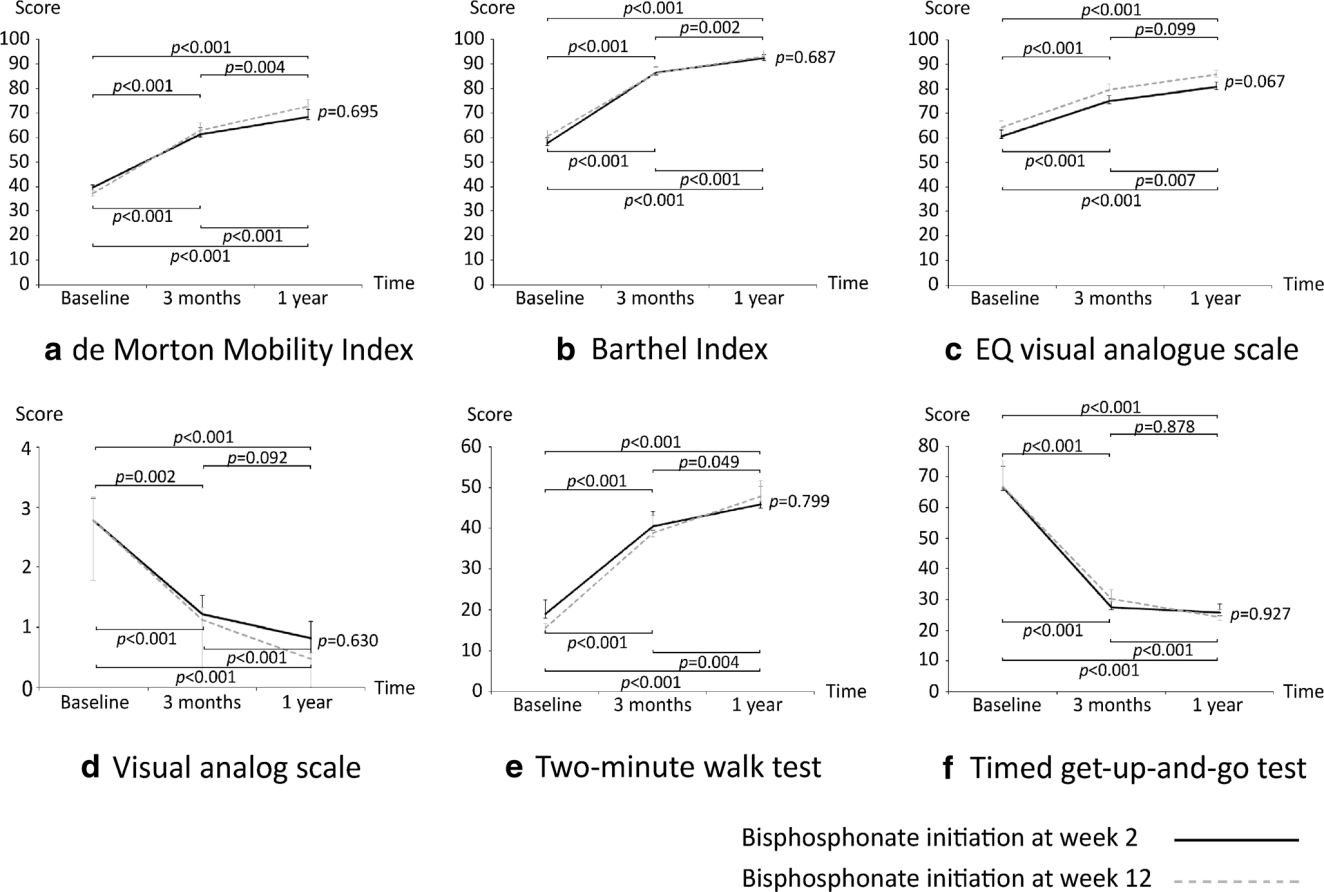
Mean change in scores from baseline to 12 months after surgery. **a** de Morton Mobility Index. **b** Barthel Index. **c** EuroQol visual analog scale. **d** Visual analog scale. **e** Two-minute walk test. **f** Timed get-up-and-go test. The *error bars* indicate the standard error. The *p* values at the top of each graph indicate the significance level of each functional outcome in the bisphosphonate initiation at week 2 group between the indicated time points. The *p* values at the bottom of each graph indicate the significance level of each functional outcome in the bisphosphonate initiation at week 12 group between the indicated time points

When evaluating functional outcomes at 1 year postoperatively, we found that all functional outcomes in the week 12 group improved significantly from those recorded at the 3-month postoperative follow-up (*p* < 0.007). In the week 2 group, only DEMMI, Barthel Index, and the 2-min walk test improved significantly from scores recorded at the 3-month postoperative follow-up (Fig. 2). There were no statistically significant differences in the improvement of functional outcomes between groups at both the 3-month and 1-year postoperative follow-ups (Fig. 2). The percentage of patients who used gait assisting device increased significantly from 27% before fracture to 60.5% at 1 year after surgery.

## Discussion

Some studies have reported that 45% or more of hip fracture patients have a prior history of fracture [8, 29], and data indicate that almost half of all women and one third of men with a hip fracture will suffer a new fragility fracture during their remaining lifetime [30, 31]. Accordingly, this patient population is a target group for osteoporosis intervention to reduce future fracture risk. It is generally accepted that bisphosphonate is the mainstay therapy for prevention and treatment of osteoporosis. However, when to start bisphosphonate treatment after a fragility fracture has not yet been definitively established.

Physicians have to consider two major issues before prescribing bisphosphonate after fracture fixation. The first issue centers on the effect that bisphosphonate will have on fracture healing. Fracture healing is a complex event that involves coordination of a variety of different processes, including inflammation, soft callus formation, hard callus formation, and remodeling and modeling [32]. Once administered, bisphosphonates bind to hydroxyapatite crystals and inhibit crystal breakdown [15]. They are preferentially incorporated into sites with active bone remodeling, and they increase apoptosis of osteoclasts, leading to diminished bone resorption. Since osteoclasts are essential for the callus remodeling process, it is hypothesized that bisphosphonate may adversely affect fracture healing. Xue et al. [33] conducted a systematic review in bisphosphonates and their influence on fracture healing and found that bisphosphonates significantly prolonged healing times in patients with distal radius fractures, but not in patients with femoral fractures. As a result of this prevailing concern regarding the effect of bisphosphonate on fracture healing, most orthopedic surgeons delay the initiation of bisphosphonate therapy for a few months after fracture fixation. The second issue relates to the potential induction of symptomatic hypocalcemia if bisphosphonate is given to those with pre-existing low serum calcium and vitamin D level [18]. For this reason, some physicians prefer to delay bisphosphonate treatment for a few months after fracture repair. In this study, we postponed bisphosphonate treatment for 3 months after fracture treatment due to the result reported from a previous investigation, which showed a 28% reduction in overall mortality in patients who received intravenous bisphosphonate within 90 days after surgical repair of an osteoporotic hip fracture [34]. Determining whether a longer delay in bisphosphonate treatment adversely affects functional recovery after hemiarthroplasty in femoral neck fracture patients would require a longer period of no bisphosphonate treatment, which could have ethical implications relative to what is considered safe and unsafe patient care.

Although guideline-recommended osteoporosis treatment with bisphosphonate can substantially reduce fracture risk to approximately 30–40% [35–37], the rate of osteoporosis treatment following hip fracture remains low [38–40]. One intervention that may improve osteoporosis treatment rates is the establishment of a fracture liaison service [41]. Another possible intervention is to encouraging physicians and orthopedic traumatologists to initiate osteoporosis treatment soon after fracture repair. Because the present study found no differences in short-term functional outcomes or significant adverse events between groups, early bisphosphonate treatment at 2 weeks after fracture fixation might be beneficial in hip fracture patients for several reasons. First, starting bisphosphonate therapy early would prevent a delay in osteoporosis treatment. Second, data from a large fracture trial showed that bisphosphonate treatment reduced fracture risk as early as 6 months after treatment initiation [42, 43]. Given that a majority of hip fracture patients are still in the hospital at the 2-week time point after fracture fixation, early bisphosphonate initiation would facilitate a closely monitored, medically supervised start to long-term osteoporosis treatment, which may improve the effectiveness of a posthip fracture care program.

It is important to emphasize that low calcium and vitamin D status must be corrected before or during the initiation of anti-osteoporosis therapy. This correction will reduce the risk of hypocalcemia and worsening secondary hyperparathyroidism that might occur if the patients are not rendered vitamin D replete prior to bisphosphonate treatment [44]. Furthermore, correction of hypovitaminosis D and hypocalcemia might enhance the response of bisphosphonate treatment [45]. Our results showed mean serum calcium level at the 3-month postoperative follow-up in the bisphosphonate initiation at week 2 group to be significantly lower than that of the week 12 group (9.1 vs. 9.4 mg/dL; *p* = 0.008). In addition, the rate of total adverse events in the week 2 group (16.3%) was higher than that of the week 12 group (5.9%). These findings can be explained by the action of the bisphosphonate in the week 2 group. Bisphosphonates are potent inhibitors of osteoclastic bone resorption. This inhibition decreases calcium efflux from bone, leading to a transient period of slight hypocalcemia. Most patients, however, do not become hypocalcemic due to compensatory mechanisms, the most important of which is increased secretion of parathyroid hormone [46]. In this study, we gave calcium and vitamin D supplementation to all patients within 2 weeks in both groups and none of them developed symptomatic hypocalcemia or discontinued the medications due to any adverse reactions. We, therefore, recommend that treating physicians correct low calcium and vitamin D levels together with the initiation of any osteoporosis drugs in order to reduce the potential side effects of osteoporotic medications.

This study has several mentionable limitations. First, the patients and physicians were not blinded, because a placebo pill was not used. Although this is a potential source of bias, our research assistants (who were unaware of the group allocations) administered the questionnaires and performed the functional outcome assessments. Second, patients in our study with no history of bisphosphonate therapy received only one type of bisphosphonate (risedronate). We are, therefore, unable to address whether patients who had prior history of bisphosphonate treatment and those who took other types of bisphosphonate would have had similar results. In addition, our findings cannot be directly extrapolated to hip fracture patients who received non-operative treatment and those without hip fracture. Third, our sample size was calculated to determine a six-point difference in DEMMI score between groups. Thus, our sample size was too small to detect other clinically important secondary outcome measures, such as visual analog scale, 2-min walk test, and adverse reactions. Furthermore, the 18.9 standard deviation of the DEMMI score found in our study at the 3-month follow-up was much higher than the 8.9 standard deviation reported in a previous investigation [20]. As previously described, a standard deviation of 8.9 was used to calculate the sample size for this study. When we used our standard deviation of 18.9 to calculate the power of the study, we found the power to be only 46. Caution should, therefore, be exercised when interpreting our results. At a standard deviation of 18.9, each group would have to have included 117 patients to achieve a power of 80. This information was not available until after the study was completed. Hence, this research should be regarded as a preliminary study that investigated the effect of bisphosphonate initiation at week 2 versus week 12 on short-term functional recovery in patients with a femoral neck fracture. Finally, the duration of follow-up in this study was relatively short. However, longer term follow-up is difficult due to a short life expectancy in this patient population [47].

In conclusion, no significant differences in short-term functional recovery or significant adverse events were observed between the week 2 and week 12 bisphosphonate initiation groups. As such, initiation of bisphosphonate therapy may be considered as early as 2 weeks after femoral neck fracture. It is important to emphasize that low serum calcium and vitamin D status must be corrected with calcium and vitamin D supplementation prior to or at the time of bisphosphonate initiation. Further studies in a larger population are needed to confirm the results of our study.

## Electronic supplementary material

ESM 1 (DOCX 29 kb).
