# Supplementary material for: Effect of bisphosphonate initiation at week 2 versus week 12 on short-term functional recovery after femoral neck fracture: a randomized controlled trial
Source: Arch Osteoporos. 2017 Mar 10;12(1):27. doi: 10.1007/s11657-017-0321-8 (PMC5346124; doi:10.1007/s11657-017-0321-8)
Supplement: Supplementary file 1 — (DOCX 29 kb). [file 11657_2017_321_MOESM1_ESM.docx]

**Supplementary Table.** Comparing data between patients who completed and did not complete the study at 1 year

| Clinical variables | Not complete  (N=19) | Complete  (N=81) | *p*-value |
| --- | --- | --- | --- |
| Age (years) | 78.8 $\pm$ 8.0 | 76.0 $\pm$ 8.2 | 0.187 |
| Female gender | 11 (57.9%) | 69 (85.2%) | 0.021 |
| Right side | 6 (31.6%) | 40 (49.4%) | 0.205 |
| Body mass index (kg/m^2^) | 23.5 $\pm$ 3.8 | 23.2 $\pm$ 4.6 | 0.803 |
| Charlson Comorbidity Index   - 0-1 - 2-3 - >3 | 16 (84.2%)  3 (15.8%)  0 (0.0%) | 70 (86.4%)  10 (12.3%)  1 (1.2%) | 0.921 |
| Cementless femoral component | 17 (89.5%) | 69 (85.2%) | 1.000 |
| Preoperative ambulatory status   - with assisting device - without assisting device | 5 (26.3%)  14 (73.7%) | 22 (27.2%)  59 (72.8%) | 1.000 |
| Estimated glomerular filtration rate  (mL/min/1.73m^2^) | 65.3 $\pm$ 21.1 | 72.9 $\pm$ 20.2 | 0.147 |
| Serum calcium level (mg/dL) | 9.1 $\pm$ 0.6 | 8.9 $\pm$ 0.5 | 0.228 |
| Serum 25(OH)D level (ng/mL) | 20.6 $\pm$ 5.7 | 22.8 $\pm$ 11.7 | 0.295 |
| Baseline bone mineral density (g/cm^2^)   - Lumbar spine - Femoral neck - Total hip | 1.019 $\pm$ 0.271  0.643 $\pm$ 0.116  0.689 $\pm$ 0.128 | 0.931 $\pm$ 0.184  0.636 $\pm$ 0.129  0.673 $\pm$ 0.122 | 0.203  0.837  0.629 |
| Data presented as number (%) or mean ± standard deviation  *p*-value<0.05 indicates statistical significance  **Abbreviations:** 25(OH)D, 25-hydroxyvitamin D  Estimated glomerular filtration rate was calculated using the Chronic Kidney Disease Epidemiology Collaboration (CKD-EPI) equation | | | |
